# Assessing Knowledge and Awareness of Scoliosis Among Adults in the Najran Region, Saudi Arabia: A Cross-Sectional Study

**DOI:** 10.7759/cureus.116794

**Published:** 2026-09-23

**Authors:** Saleh Kardm, Ammar M Alrashah, Alhussain Algodrah, Khaled Al Agail, Abdullah Alqabqab, Abdullah Al-Sharif, Ali Alharshan, Abdullah Asiri, Mohammed Al-Hareth, Dhafer Alkhudhrah

**Affiliations:** 1 Department of Surgery, Najran University, Najran, SAU

**Keywords:** knowledge level, najran, public awareness, saudi arabia, scoliosis

## Abstract

Background: Scoliosis is a three-dimensional spinal deformity that may affect posture, function, and, in severe cases, cardiopulmonary function. Community knowledge may contribute to early recognition, appropriate clinical assessment, and timely referral. This study aimed to describe item-level knowledge and awareness of scoliosis among adults recruited in the Najran region and to explore associations between the investigator-defined knowledge score and selected measured participant characteristics.

Methods: A cross-sectional study was conducted among adults recruited in the Najran region, Saudi Arabia, using a self-administered online questionnaire. Non-probability convenience sampling was used. Of 508 collected responses, 39 respondents who reported not residing in the Najran region were excluded in accordance with the eligibility criteria, resulting in a final analytic sample of 469 participants. The investigator-developed questionnaire was reviewed by a consultant pediatric orthopedic surgeon and pilot-tested among 20 individuals who were not included in the final analysis. An investigator-defined knowledge score was calculated, and a score of >50% (≥10/18 points) was operationally classified as good knowledge.

Results: A total of 469 eligible participants were analyzed. Most participants had a university education (69.7%), and 13.2% reported a family history of scoliosis. Correct identification of scoliosis as a deviation of the spine from its normal position was reported by 77.8% of participants, while 67.0% identified radiographic imaging as a diagnostic method. The investigator-defined knowledge score had a median of 8 (IQR, 5-11), with observed scores ranging from 0 to 17. Using the exploratory >50% cutoff (≥10/18 points), 179 participants (38.2%) were categorized as having good knowledge and 290 (61.8%) as having poor knowledge. No statistically significant associations were detected between knowledge category and the measured participant characteristics.

Conclusions: Among the surveyed participants, important item-level gaps in knowledge and awareness of scoliosis were identified. The investigator-defined composite knowledge classification should be interpreted cautiously because the questionnaire and scoring threshold were not formally validated. The findings identify potential targets for educational initiatives, while future studies using validated instruments and more representative sampling methods are needed to confirm these findings.

## Introduction

Scoliosis is a three-dimensional spinal deformity characterized by abnormal curvature of the spine. On standing coronal radiographs, scoliosis is typically defined by a Cobb angle of at least 10° [1,2]. The epidemiology of scoliosis varies considerably according to age, scoliosis type, study population, and method of ascertainment. A systematic review of adult de novo scoliosis reported a pooled prevalence estimate of 37.6% (95% confidence interval (CI), 18.7%-61.8%), with higher estimates among older adults [3]. In children and adolescents, a systematic review reported a prevalence of approximately 3.1% [4], while a population-wide analysis from mainland China reported a prevalence of 1.20% among adolescents [5].

In Saudi Arabia, reported estimates have also varied according to the population studied and the screening or diagnostic method used. A radiology-based study in Taif reported scoliosis as the most common spinal disorder in its study sample [6]. Studies conducted in Riyadh and Abha have reported suspected or detected scoliosis among adolescents [7,8], whereas the Saudi National School-Based Screening Program reported a substantially lower prevalence of 0.48% among primary school students [9]. These variations highlight the importance of interpreting prevalence estimates within the specific age group, clinical context, and screening methodology rather than considering scoliosis as a homogeneous condition across populations.

Community understanding of scoliosis is relevant because recognition of spinal deformity and awareness of appropriate evaluation may facilitate early recognition, appropriate clinical assessment, and timely referral. A previous Saudi study reported substantial gaps in public knowledge and awareness of scoliosis [10]. In addition, parents of children diagnosed with adolescent idiopathic scoliosis may experience unmet information needs and concerns regarding treatment and psychosocial effects [11]. However, region-specific evidence regarding community knowledge and awareness of scoliosis in the Najran region remains limited. Identifying specific misconceptions and knowledge gaps may help define potential targets for future educational initiatives.

Therefore, this study aimed to describe item-level knowledge and awareness of scoliosis among adults recruited in the Najran region and to explore associations between the investigator-defined knowledge score and selected measured participant characteristics.

## Materials and methods

Study design and setting

A cross-sectional study was conducted among adults recruited in the Najran region, Saudi Arabia. The study was designed to assess item-level knowledge and awareness regarding scoliosis and to explore associations between an investigator-defined knowledge score and selected measured participant characteristics. Data collection commenced after obtaining ethical approval from the Institutional Review Board.

Study population and eligibility criteria

Adults aged 18 years or older who reported residing in the Najran region and agreed to participate were eligible for inclusion. Residence in the Najran region was determined by participants’ self-report through the first questionnaire item.

A total of 508 responses were available in the original dataset. During verification of the dataset for the revised analysis, 39 respondents were identified as having reported that they were not residents of the Najran region and were therefore excluded in accordance with the eligibility criteria. The final analytic sample consisted of 469 eligible participants.

Sampling and data collection

A non-probability convenience sampling technique was used. Data were collected using a self-administered online questionnaire administered through Google Forms. The survey link was distributed through WhatsApp and Telegram using a non-probability convenience sampling approach.

Participation was voluntary. To minimize duplicate participation, the “Limit to 1 response” setting in Google Forms was enabled, requiring participants to sign in to a Google account before submitting the questionnaire and restricting each signed-in account to a single submission.

Because the survey invitation was distributed openly through social media and the total number of individuals who received or accessed the invitation was not available, a conventional response rate could not be calculated.

Questionnaire development and pilot testing

The questionnaire was investigator-developed and reviewed by a consultant pediatric orthopedic surgeon to assess the relevance, clarity, and clinical appropriateness of the questionnaire items. This expert review was used to assess the content and face appropriateness of the instrument; however, no formal quantitative content-validity assessment was performed. A pilot study was subsequently conducted among 20 individuals to assess the clarity and comprehensibility of the questionnaire. Participants involved in the pilot study were not included in the final study sample or statistical analysis. The questionnaire did not undergo formal psychometric validation or internal consistency reliability testing.

The questionnaire was administered to participants in Arabic. For presentation in the manuscript, it was translated into English by the consultant pediatric orthopedic surgeon who reviewed the instrument (Appendix). A formal forward-backward translation procedure was not performed. Formal psychometric validation and internal consistency reliability testing were not performed.

The questionnaire included items assessing educational level, family history of scoliosis, self-rated knowledge, knowledge of the definition, possible causes, risk groups, diagnosis, clinical manifestations, treatment, and complications of scoliosis, as well as previous participation in awareness activities, perceived community awareness, support for awareness initiatives, and sources of information. Some knowledge questions permitted selection of more than one response.

Knowledge scoring

An investigator-defined scoring system was used to summarize responses to the knowledge items. One point was assigned for each response designated as correct in the study scoring key, whereas incorrect responses and “I do not know” responses received zero points. For multiple-response questions, one point was awarded for each designated correct response selected; selection of an incorrect response did not result in point deduction.

After clarification of the scoring algorithm according to the structure of the questionnaire and the original investigator-defined scoring key, the maximum achievable knowledge score was 18 points. For descriptive and exploratory purposes, a threshold of >50% of the maximum achievable score was used to categorize participants; therefore, scores of ≥10/18 points were operationally classified as good knowledge, whereas scores of ≤9/18 points were classified as poor knowledge. This threshold was selected by the investigators as a simple operational cutoff for summarizing the study responses and was not derived from a previously validated instrument or an established clinically meaningful threshold. Accordingly, the resulting good/poor knowledge categories should not be interpreted as validated classifications of participants’ knowledge. The categorical analysis was treated as exploratory, and item-level responses were emphasized to allow interpretation of the findings independently of the composite score.

Statistical analysis

Data were analyzed using SPSS Statistics (IBM Corp., Armonk, NY, USA). Categorical variables were summarised as frequencies and percentages. The distribution of the investigator-defined knowledge score was assessed using the Shapiro-Wilk test. A P-value <0.05 was considered evidence of a statistically significant departure from normality.

Associations between the categorical knowledge classification and measured participant characteristics were evaluated using the Pearson chi-square test when its assumptions were satisfied and an exact test when expected cell counts were insufficient. Effect estimates were reported where appropriate, including odds ratios with 95% confidence intervals (CIs) for binary comparisons and Cramér’s V for categorical variables with more than two levels. A two-sided P-value <0.05 was considered statistically significant.

Ethical considerations

Ethical approval was obtained from the Institutional Review Board of King Khalid Hospital, Najran Health Cluster, Saudi Arabia (Approval No. IRB Log Mar 2026-94A). The study was conducted in accordance with the ethical principles of the Declaration of Helsinki. Electronic informed consent was obtained from all participants before participation. Participation was voluntary, and confidentiality and anonymity were maintained throughout the study.

## Results

Participant characteristics

A total of 508 responses were available in the original dataset. After applying the residence eligibility criterion, 39 respondents who reported that they were not residents of the Najran region were excluded, resulting in a final analytic sample of 469 participants.

Most participants had university-level education (327, 69.7%), followed by primary, secondary, or high school education (112, 23.9%), postgraduate education (28, 6.0%), and no formal education (2, 0.4%). A family history of scoliosis was reported by 62 participants (13.2%), whereas 407 (86.8%) reported no family history (Table 1).

**Table 1:** Characteristics of the study participants (N = 469)

Characteristic	n	%
Educational level		
Illiterate	2	0.4
Primary/secondary/high school education	112	23.9
University education	327	69.7
Postgraduate education	28	6.0
Family history of scoliosis		
Yes	62	13.2
No	407	86.8

Item-level knowledge of scoliosis

Most participants (365, 77.8%) identified scoliosis as a deviation of the spine from its normal position. Regarding possible causes, 134 (28.6%) selected a family genetic factor, 118 (25.2%) selected an unknown cause, 49 (10.4%) selected genetic mutations, and 39 (8.3%) selected injuries and accidents. In addition, 86 participants (18.3%) reported that they did not know the cause.

Regarding groups perceived to be at higher risk, 193 participants (41.2%) selected both males and females equally, 108 (23.0%) selected females, 93 (19.8%) selected males, and 75 (16.0%) responded that they did not know. For age groups, 91 participants (19.4%) selected 10-20 years, while 94 (20.0%) selected adults older than 30 years.

For diagnosis, radiographic imaging was the most frequently selected response (314, 67.0%), followed by clinical examination (223, 47.5%) and medical history (157, 33.5%). Laboratory samples were selected by 72 (15.4%), and 59 (12.6%) responded that they did not know. Multiple selections were permitted for this item.

The most frequently selected clinical manifestations were back pain (278, 59.3%), visible spinal curvature (247, 52.7%), and abnormal body posture (190, 40.5%). Numbness or tingling in the lower extremities was selected by 75 (16.0%), while 21 (4.5%) selected the possibility of no prominent or obvious symptoms.

Regarding treatment, surgical correction and fixation was selected by 214 participants (45.6%), physical therapy and stretching exercises by 184 (39.2%), and bracing by 164 (35.0%). Regarding potential complications, 295 (62.9%) selected increased spinal deviation affecting body appearance, 265 (56.5%) selected vertebral compression with impaired mobility, 218 (46.5%) selected gait imbalance or difficulty in movement, and 96 (20.5%) selected cardiopulmonary impairment (Table 2).

**Table 2:** Item-level knowledge of scoliosis among study participants (N = 469) Response designated as correct in the original investigator-defined scoring key. Multiple responses were permitted; therefore, percentages may not sum to 100%.

Question/response option	n	%
What is scoliosis?		
A deformity that causes swelling and chronic pain in the joints	16	3.4
Bacterial infection of internal organs	19	4.1
Deviation of the spine from its normal position*	365	77.8
Genetic disease involving allergies and skin rashes	8	1.7
I do not know	61	13.0
What are the causes of scoliosis?		
Family genetic factor*	134	28.6
Genetic mutations*	49	10.4
Injuries and accidents*	39	8.3
Poor nutrition	7	1.5
Poor physical fitness	36	7.7
Unknown cause*	118	25.2
I do not know	86	18.3
Who is more likely to develop scoliosis?		
Males	93	19.8
Females*	108	23.0
Both equally	193	41.2
I do not know	75	16.0
Which age group is most at risk of developing scoliosis?		
Newborns	83	17.7
Children under 10 years	70	14.9
10-20 years*	91	19.4
20-30 years	44	9.4
Adults over 30 years	94	20.0
I do not know	87	18.6
How is scoliosis diagnosed?		
Medical history*	157	33.5
Clinical examination*	223	47.5
Radiographic imaging*	314	67.0
Laboratory samples	72	15.4
I do not know	59	12.6
What are the symptoms/clinical manifestations of scoliosis?		
Back pain*	278	59.3
Inability to walk and move	117	24.9
Back swelling	111	23.7
Abnormal body posture	190	40.5
Visible spinal curvature*	247	52.7
Paralysis of the upper and lower limbs	73	15.6
Numbness and tingling in the lower extremities*	75	16.0
Colored spots on the skin	37	7.9
No prominent or obvious symptoms*	21	4.5
I do not know	72	15.4
What are the treatments for scoliosis?		
Palliative psychotherapy only	25	5.3
Physical therapy and stretching exercises*	184	39.2
Special belts and a back brace*	164	35.0
Surgical correction and fixation*	214	45.6
There is no guaranteed treatment at the present time	50	10.7
All of the above	168	35.8
What are the complications/consequences of untreated scoliosis?		
Increased spinal deviation affecting body appearance*	295	62.9
Compression of the vertebrae resulting in loss or impairment of mobility*	265	56.5
Cardiopulmonary impairment*	96	20.5
Gait imbalance and difficulty in movement*	218	46.5
I do not know	73	15.6

Self-rated knowledge, awareness, and sources of information

Regarding self-rated knowledge of scoliosis, 235 participants (50.1%) rated their knowledge as poor, 164 (35.0%) as moderate, and 70 (14.9%) as good.

Only 41 participants (8.7%) reported having previously attended a scoliosis awareness campaign or activity. Regarding community awareness in the Najran region, 246 (52.5%) considered awareness insufficient, 54 (11.5%) considered it sufficient, and 169 (36.0%) responded that they did not know.

Most participants (422, 90.0%) supported organizing scoliosis awareness exhibitions in schools, universities, and public places. Social media was the most frequently reported source of information (179, 38.2%), followed by other sources (140, 29.9%), healthcare providers (102, 21.7%), and family or friends (48, 10.2%) (Table 3).

**Table 3:** Self-rated knowledge, awareness, and sources of information regarding scoliosis among study participants (N = 469)

Characteristic	n	%
Self-rated knowledge of scoliosis		
Poor	235	50.1
Moderate	164	35.0
Good	70	14.9
Previously attended a scoliosis awareness campaign or activity		
Yes	41	8.7
No	428	91.3
Perception of sufficient community awareness about scoliosis in the Najran region		
Yes	54	11.5
No	246	52.5
I do not know	169	36.0
Support for organizing awareness exhibitions in schools, universities, and public places		
Yes	422	90.0
No	47	10.0
Source of information about scoliosis		
Family/friends	48	10.2
Healthcare providers	102	21.7
Social media	179	38.2
Other	140	29.9

Investigator-defined knowledge score

Using the scoring key documented in the study instrument, the investigator-defined knowledge score had a theoretical range of 0-18 points. Observed scores ranged from 0 to 17, with a mean score of 7.79 ± 4.29 and a median of 8 (IQR, 5-11).

The knowledge score distribution showed a statistically significant departure from normality on the Shapiro-Wilk test (W = 0.965, P < 0.001). Accordingly, the median and interquartile range are emphasized when describing the distribution of the knowledge score.

Using the >50% threshold (≥10/18 points), 179 participants (38.2%; 95% CI, 33.9%-42.6%) were categorized as having good knowledge, whereas 290 (61.8%; 95% CI, 57.4%-66.1%) were categorized as having poor knowledge. This exploratory classification should be interpreted alongside the item-level findings.

Associations between measured participant characteristics and knowledge classification

No statistically significant associations were detected between the investigator-defined knowledge category and the measured participant characteristics. These analyses were exploratory and were limited to characteristics collected in the questionnaire. Because participant age and sex were not collected, adjustment or stratification for these potentially relevant demographic characteristics was not possible. Accordingly, the observed associations should be interpreted cautiously and should not be considered independent of potential confounding by unmeasured age and sex.

Educational level was not significantly associated with knowledge classification (Fisher-Freeman-Halton exact test, P = 0.304; Cramér’s V = 0.082). Family history of scoliosis was also not significantly associated with knowledge classification (Pearson’s χ²(1) = 0.430, P = 0.512; OR = 1.20, 95% CI 0.70-2.06).

Previous attendance at a scoliosis awareness activity was not significantly associated with knowledge classification (Pearson’s χ²(1) = 0.794, P = 0.373; OR = 0.73, 95% CI 0.37-1.46). Perception of sufficient community awareness was also not significantly associated with knowledge classification (Pearson’s χ²(2) = 4.488, P = 0.106; Cramér’s V = 0.098).

Support for organizing scoliosis awareness exhibitions showed a positive but statistically non-significant association with good knowledge (Pearson’s χ²(1) = 3.533, P = 0.060; OR = 1.91, 95% CI 0.96-3.79) (Table 4).

**Table 4:** Association between measured participant characteristics and investigator-defined knowledge classification (N = 469) Values are presented as n (row %). Fisher-Freeman-Halton exact test was used for educational level because of small expected cell counts. Pearson’s chi-square test was used for the remaining comparisons. A two-sided P-value <0.05 was considered statistically significant.

Characteristic	Poor knowledge, n (%)	Good knowledge, n (%)	P-value
Educational level	0.304*.		
Illiterate	1 (50.0)	1 (50.0)	
Primary/secondary/high school education	77 (68.8)	35 (31.2)	
University education	196 (59.9)	131 (40.1)	
Postgraduate education	16 (57.1)	12 (42.9)	
Family history of scoliosis	0.512		
No	254 (62.4)	153 (37.6)	
Yes	36 (58.1)	26 (41.9)	
Previously attended a scoliosis awareness activity	0.373		
No	262 (61.2)	166 (38.8)	
Yes	28 (68.3)	13 (31.7)	
Perception of sufficient community awareness	0.106		
No	152 (61.8)	94 (38.2)	
Yes	40 (74.1)	14 (25.9)	
I do not know	98 (58.0)	71 (42.0)	
Support for organizing awareness exhibitions	0.060		
No	35 (74.5)	12 (25.5)	
Yes	255 (60.4)	167 (39.6)	

## Discussion

This cross-sectional study assessed scoliosis knowledge and awareness among adults recruited in the Najran region of Saudi Arabia. The findings demonstrated important item-level variation in participants’ understanding of scoliosis. Although 77.8% correctly identified scoliosis as a deviation of the spine from its normal position and 67.0% recognized radiographic imaging as a diagnostic modality, knowledge was less consistent across other aspects, including possible causes, risk groups, clinical manifestations, and management. The exploratory knowledge classification categorized 61.8% of participants as having poor knowledge and 38.2% as having good knowledge. Given the limitations of the composite score, the item-level findings provide the more informative basis for interpreting specific knowledge gaps.

The observed knowledge gaps are consistent with the broader concern that public awareness of scoliosis in Saudi Arabia remains incomplete [10]. Comparisons with previous studies should, however, be interpreted cautiously because study populations, questionnaires, scoring systems, and sampling methods differ. For example, awareness among schoolteachers may differ from that of the general adult population because of differences in educational and professional exposure [12]. International studies involving students and teachers have likewise demonstrated variable levels of scoliosis awareness [13,14].

Although 77.8% of participants recognized the basic definition of scoliosis, responses concerning risk groups and clinical manifestations were more heterogeneous. Only 23.0% selected females as being more likely to develop scoliosis, while 41.2% selected both sexes equally. Similarly, 19.4% selected the 10-20-year age group as being at greatest risk. These responses should not be interpreted simply as correct or incorrect across all forms of scoliosis because age and sex distributions vary according to scoliosis type and clinical context. Regarding clinical manifestations, back pain, visible spinal curvature, and abnormal body posture were selected by 59.3%, 52.7%, and 40.5% of participants, respectively. Clinical literature describes spinal deformity and asymmetry as important features, while pain may be particularly relevant in adults [15,16].

Knowledge of scoliosis management was also variable. Surgical correction and fixation was selected by 45.6% of participants, physical therapy and stretching exercises by 39.2%, and bracing by 35.0%. Management of scoliosis depends on factors such as scoliosis type, age, curve magnitude and progression, symptoms, and skeletal maturity, and treatment options are therefore not universally applicable to all patients [16]. These findings highlight the importance of educational information that presents scoliosis management as individualized rather than implying that a single intervention is appropriate for all patients.

A notable finding was the mismatch between limited previous exposure to formal awareness activities and strong support for future initiatives. Only 8.7% of participants had previously attended a scoliosis awareness campaign or activity, whereas 90.0% supported organizing awareness exhibitions in schools, universities, and public places. Social media was the most commonly reported source of scoliosis information (38.2%). This may provide an accessible channel for community education; however, the accuracy and quality of online scoliosis information require consideration because social-media content may vary substantially in reliability [17].

No statistically significant associations were detected between the investigator-defined knowledge category and the measured participant characteristics. Educational level, family history of scoliosis, previous attendance at awareness activities, perception of sufficient community awareness, and support for awareness exhibitions did not reach statistical significance. Support for awareness exhibitions showed a positive but statistically non-significant association with good knowledge (OR = 1.91, 95% CI: 0.96-3.79; P = 0.060). The absence of statistically significant associations should not be interpreted as evidence that these characteristics have no relationship with scoliosis knowledge, particularly given the convenience-sampling design, small numbers in some categories, and uncertainty reflected in the confidence intervals.

Strengths and limitations

This study provides region-specific information on item-level scoliosis knowledge and awareness among adults recruited in the Najran region, and the final analytic sample of 469 participants exceeded the calculated minimum sample size. Nevertheless, several limitations should be considered. First, the cross-sectional design does not permit causal inference. Second, non-probability convenience sampling and recruitment through social media may have introduced selection and digital-access bias, thereby limiting the generalizability of the findings. This potential selection bias is also reflected in the educational profile of the sample, as 69.7% of participants had a university education, which may limit the generalizability of the findings to the broader adult population of Najran. The total number of individuals who received or accessed the survey invitation was not available; therefore, a conventional response rate could not be calculated. To reduce the risk of duplicate responses, the Google Forms survey was configured to allow only one submission per signed-in account. However, this measure could not exclude the possibility that different accounts were used by the same individual.

Third, participant age and sex were not collected in the questionnaire. Consequently, the demographic distribution of the sample with respect to these characteristics could not be described, and knowledge scores could not be stratified or adjusted according to age or sex. This is an important limitation because age and sex may be associated with differences in exposure to scoliosis-related information and knowledge. Therefore, the observed associations between the measured participant characteristics and the investigator-defined knowledge classification should be interpreted cautiously, as potential confounding by age and sex could not be assessed. Future studies should collect these demographic variables and incorporate them into adjusted analyses.

Finally, the questionnaire was administered in Arabic, while the English version presented in the manuscript was translated by the consultant pediatric orthopedic surgeon who reviewed the instrument; a formal forward-backward translation procedure was not performed. Self-reported responses may also be affected by recall or response bias.

## Conclusions

This study identified important item-level gaps in scoliosis knowledge and awareness among adults recruited in the Najran region. Although most participants recognized the basic definition of scoliosis and radiographic imaging as an important diagnostic modality, knowledge varied across possible causes, risk groups, clinical manifestations, and management. The exploratory classification categorized 61.8% of participants as having poor knowledge and 38.2% as having good knowledge. These findings should be considered alongside the item-level responses, which provide a more detailed representation of specific areas of knowledge and awareness. The low level of previous participation in awareness activities and the high level of support for future awareness initiatives identify potential targets for community education. Future studies using validated instruments, more representative sampling methods, and broader demographic characterization are needed to confirm these findings and evaluate the effectiveness of targeted awareness initiatives.

## Appendices

Questionnaire (English translation)

**Table 5:** English Translation of the Study Questionnaire Note: The questionnaire was administered in Arabic and translated into English for manuscript reporting by a consultant pediatric orthopedic surgeon.

No.	Questionnaire item	Response options
1	Are you a resident of the Najran region?	Yes; No
2	Educational level	Illiterate; Primary/secondary/high school education; University education; Postgraduate education
3	What is scoliosis?	A deformity that causes swelling and chronic pain in the joints of the body; Bacterial infection of internal organs; Deviation of the spine from its normal position; Genetic disease involving allergies and skin rashes; I do not know
4	Is there a family history of scoliosis (spinal curvature)?	Yes; No
5	How would you rate your knowledge of scoliosis (spinal curvature)?	Poor; Moderate; Good
6	What are the causes of scoliosis?	Family genetic factor; Genetic mutations; Injuries and accidents; Poor nutrition; Poor physical fitness; Unknown cause; I do not know
7	In your opinion, who is more likely to develop scoliosis?	Males; Females; Both equally; I do not know
8	Which age groups are most at risk of developing scoliosis?	Newborns; Children under 10 years of age; 10-20 years; 20-30 years; Adults over 30 years of age; I do not know
9	How is scoliosis diagnosed? (More than one option may be selected)	Medical history; Clinical examination; Radiographic imaging; Laboratory samples; I do not know
10	What are the symptoms of scoliosis? (More than one option may be selected)	Back pain; Inability to walk and move; Back swelling; Abnormal body posture; Visible spinal curvature; Paralysis of the upper and lower limbs; Numbness and tingling in the lower extremities; Colored spots on the skin; No prominent or obvious symptoms; I do not know
11	What are the treatments for scoliosis? (More than one option may be selected)	Palliative psychotherapy only; Physical therapy and stretching exercises for the back muscles; Special belts and a back brace; Surgical treatment by correction and fixation; There is no guaranteed treatment at the present time; All of the above
12	What are the complications and consequences of failure to detect or treat scoliosis? (More than one option may be selected)	Increased spinal deviation affecting body appearance; Compression of the vertebrae resulting in loss or impairment of mobility; Cardiopulmonary impairment; Gait imbalance and difficulty in movement; I do not know
13	Have you previously attended any scoliosis awareness campaigns or activities?	Yes; No
14	Do you think there is sufficient community awareness about scoliosis in the Najran region?	Yes; No; I do not know
15	Do you support organizing awareness exhibitions in schools, universities, and public places to increase awareness of scoliosis?	Yes; No
16	What is your source of information about scoliosis (spinal curvature)?	Family/friends; Healthcare providers; Social media; Other
